# A Human-Centered Multimodal Framework for Characterizing Safety-Relevant Driver Functional Domains: An Exploratory Study of Professional Bus Drivers †

**DOI:** 10.3390/s26123664

**Published:** 2026-06-08

**Authors:** Ting-An Kuo, Chiuhsiang Joe Lin, Po-Hsiang Liu

**Affiliations:** 1 Department of Industrial Management, National Taiwan University of Science and Technology, Taipei 10607, Taiwan; a76526@gmail.com; 2 Department of Industrial Management, St. John’s University, New Taipei City 25135, Taiwan; bsliu@mail.sju.edu.tw

**Keywords:** professional bus drivers, human-performance assessment, safety-relevant functional domains, cognitive–perceptual performance, psychomotor performance, hazard perception, visual tracking, self-perception, human-centered framework, driver monitoring research, intelligent transportation systems

## Abstract

**Highlights:**

This study proposes a human-centered framework for characterizing safety-relevant driver functional domains by integrating self-perception, psychomotor performance, and cognitive–perceptual assessment. The findings suggest that multidimensional human-performance measures may help reveal meaningful differences in driver-related functional capacities and may provide candidate constructs for future driver monitoring research.

**What are the main findings?**

- Multidimensional human-performance measures revealed selected age- and gender-related differences and descriptive tendencies across self-perception, psychomotor, and cognitive–perceptual domains.
- Older drivers showed slower and less efficient performance tendencies in several cognitive–perceptual tasks, with the clearest age-related pattern observed in the tachistoscopic traffic test.

**What are the implications of the main findings?**

- The proposed framework highlights safety-relevant human-factor domains that may serve as candidate target constructs for future driver monitoring research.
- The findings support a more explainable and human-centered perspective on driver-related assessment in intelligent transportation systems, beyond surface-level symptom detection alone.

**Abstract:**

This study proposes a human-centered multimodal framework for characterizing safety-relevant driver functional domains in professional bus drivers. Unlike conventional approaches that rely on isolated psychological or physical assessments, the proposed framework integrates self-perception, psychomotor performance, and cognitive–perceptual assessment to provide an exploratory, structured characterization of driver-related functional capacities. Eighteen professional bus drivers participated in this study. Self-perception data were obtained from all 18 participants, whereas psychomotor and cognitive–perceptual assessments were completed by 16 participants. These measurements were used to examine multiple domains relevant to driving safety, including behavioral awareness, motor coordination, attention, visual tracking, and hazard-perception-related processing. Given the modest sample size, the study should be regarded as an exploratory pilot investigation. Data were analyzed using a laboratory-based cross-sectional between-subjects design to examine age- and gender-related differences across the assessed domains. The findings suggested that selected age- and gender-related differences and descriptive tendencies were observable across multiple domains. Male drivers descriptively showed higher self-rating scores, female drivers showed different performance tendencies in selected psychomotor tasks, and male drivers demonstrated substantially greater grip strength. Older drivers showed slower and less efficient performance in several cognitive–perceptual measures, with the clearest age-related effect observed in the tachistoscopic traffic test, where older participants showed a higher error tendency under time-constrained traffic-scene processing conditions. The constructs and measures proposed in this study are intended as general laboratory-based assessments of driver-related capabilities rather than direct measures of actual driving performance, real-time driver-state indicators, or validated sensor-based monitoring indicators. As candidate human-factor constructs, they may inform future driver monitoring research by helping clarify how driver-related signals or behaviors could eventually be linked to underlying functional and safety-related meaning in intelligent transportation environments.

## 1. Introduction

Recent advances in sensing technologies have significantly transformed the way human performance is monitored in complex operational environments. In intelligent transportation systems, driver monitoring systems (DMS) have emerged as a critical application of human-centered sensing, integrating multimodal data—such as visual behavior, cognitive responses, and physiological signals—to assess driver state in real time. These systems aim to detect functional impairments, including fatigue, inattention, and reduced hazard perception, which are major contributors to road accidents. Recent driver-monitoring research has increasingly emphasized multimodal sensing approaches that combine visual, behavioral, and physiological indicators to estimate driver state. For example, physiological measures such as EEG, ECG, and electrodermal activity have been used to assess driver attention in simulated manual and automated driving scenarios [1]. In addition, physiological and eye-tracking metrics have been systematically examined as indicators of driver cognitive load [2], and multimodal physiological datasets integrating EEG, ECG, EMG, GSR, and eye-movement data have been developed for driving behavior research [3]. Camera-based approaches using facial landmarks, head posture, and eye-area features have also been proposed for real-time driver monitoring [4]. These studies indicate that driver monitoring is increasingly moving toward multimodal and interpretable sensing approaches.

Road safety has long been recognized as a major global public health concern. According to the World Health Organization, more than 1.19 million people die annually in traffic crashes worldwide [5]. Human factors are the dominant contributors to these incidents, accounting for over 90% of crashes in classical and contemporary studies [6,7]. Consequently, research has increasingly emphasized the importance of evaluating driver-related characteristics, including cognitive, perceptual, and behavioral factors [8,9].

Occupational drivers, such as bus operators, represent a particularly critical group due to their elevated exposure to traffic risks. Work-related road crashes account for 20–40% of occupational fatalities in industrialized countries [10], and commercial vehicles exhibit significantly higher fatality rates compared to private vehicles [11]. This issue is especially pronounced in aging societies, where public transportation systems rely heavily on older drivers [12,13,14]. Age- and gender-related differences have been widely investigated as key determinants of driving performance. Previous studies have shown that aging is associated with declines in reaction time, visual acuity, and motor coordination [15,16], while gender differences are often reflected in risk-taking behavior and psychomotor performance [17,18]. These findings highlight the need for comprehensive evaluation frameworks that can capture multiple dimensions of driver capability.

In parallel, psychomotor and cognitive assessment tools have been developed to quantify driver-related functional abilities. Systems such as the Vienna Test System provide objective measures of attention, perceptual speed, and decision-making under time constraints [19]. Complementary tests, including hand–eye coordination, grip strength, and balance assessments, have also been widely used to evaluate neuromuscular performance relevant to driving tasks [8,20,21]. In addition, self-assessment tools, such as the American Automobile Association (AAA) questionnaire, provide insights into drivers’ perceived competence and behavioral tendencies [22,23].

Despite these advances, a critical limitation remains in the context of driver monitoring research: the lack of a structured mapping between experimentally measured human-performance variables and the underlying functional domains that future monitoring systems may seek to estimate. Existing driver monitoring system (DMS) approaches often rely on observable or data-driven features without explicitly linking these signals to validated human functional domains, which limits their interpretability and generalizability across different driver populations.

In the present study, driver functional state is defined as a multidimensional profile of driver-related human capacities that may influence safety-relevant driving behavior, including self-perceptual awareness, psychomotor coordination, biomechanical capability, sustained attention, visual tracking, and rapid traffic-scene processing. This concept is broader than a single transient state such as fatigue, workload, or momentary attention. It also differs from general fitness-to-drive, which is typically used as a broader clinical, licensing, or regulatory judgment regarding whether a driver is suitable to operate a vehicle. In contrast, the present framework uses laboratory-based human-performance measures to characterize candidate functional domains that may be relevant to future driver monitoring research. Therefore, constructs such as attention, visual tracking, and hazard perception are treated here as specific components of driver functional state rather than as equivalent concepts. This definition is also consistent with recent driver-monitoring studies showing that driver state can be reflected through multiple observable channels, including ocular behavior, physiological responses, vehicle-control behavior, and task performance [1,2,3,4]. However, these observable signals require an interpretable human-factor framework to clarify which underlying functional domains they may represent. Therefore, the present study emphasizes the conceptual linkage between laboratory-based human-performance indicators and candidate functional domains that may inform future sensing-based driver monitoring. To address this issue, the present study proposes a human-centered multimodal framework for driver evaluation that integrates multiple domains of human performance. Specifically, the framework incorporates (a) self-perception assessment to capture subjective awareness and behavioral tendencies, (b) psychomotor assessment to evaluate neuromuscular and coordination performance, and (c) cognitive–perceptual assessment to examine attention, visual tracking, and hazard perception. By organizing these components within a unified framework, the proposed approach provides an exploratory and more comprehensive characterization of safety-relevant driver functional domains.

Parts of the assessment results reported in this study are related to two of our previous conference papers, which separately presented different components of the same participant cohort and measurement dataset [24,25]. One conference paper primarily focused on self-rating and psychomotor-related assessments, whereas the other primarily focused on cognitive–perceptual assessments using the Vienna Test System. In the present study, these previously separated components are reorganized and reinterpreted within a unified human-centered framework. The novelty of the present manuscript lies not simply in combining previously reported measures, but in providing a cross-domain interpretive structure, a more explicit characterization of safety-relevant driver functional domains, and a clearer discussion of how these experimentally measured variables may serve as candidate constructs for future driver monitoring research.

Using a sample of professional bus drivers, this study applies the proposed framework to examine how age and gender influence self-perception, psychomotor performance, and cognitive–perceptual performance. Beyond reporting these human-performance measurements, the present study seeks to clarify which domains of driver functioning may be most relevant to driving safety and how they may be interpreted as meaningful candidate target constructs for future intelligent driver monitoring systems. In this way, the study extends prior human-factor-oriented reports by providing an integrated human-centered framework that helps connect monitored driver-related signals or behaviors to their underlying functional and safety-related meaning, thereby supporting a more explainable and application-oriented perspective on driver-state interpretation in intelligent transportation environments.

## 2. Methods

### 2.1. Participants

Eighteen professional bus drivers were recruited from a major urban transportation company in Taipei, Taiwan. The sample included 11 male and 7 female drivers, all of whom held valid commercial driving licenses and had at least six years of professional driving experience. Participants reported no history of neurological disorders, uncorrected visual impairments, or musculoskeletal injuries that could affect task performance. All participants provided informed consent prior to participation.

Participant characteristics were summarized using demographic and occupational background information. For the full sample of 18 participants, the mean height was 166.3 cm (SD = 7.6, range = 156–180 cm), and the mean body weight was 70.0 kg (SD = 10.4, range = 50–90 kg). The sample included drivers from three age categories: 30–39, 40–49, and 50–59 years. Regarding professional driving experience, 13 participants had 6–10 years of experience, and 5 participants had more than 10 years of experience. Most participants reported driving more than 8 h per day, with 17 participants in this category and 1 participant reporting 6–8 h per day.

Because data availability differed across assessment components, the effective sample size varied by measurement module. AAA self-rating data were available for all 18 participants. In contrast, psychomotor and cognitive–perceptual analyses were based on 16 participants because the performance-test data of two participants were not considered valid for analysis and were therefore excluded from those modules. Specifically, one male participant and one female participant were excluded from the performance-based analyses because the Vienna Test System assessments were not completed correctly, resulting in abnormal test records. For the 16 participants included in the performance-based analyses, the valid sample included 10 male and 6 female drivers. Regarding professional driving experience, 12 participants had 6–10 years of experience, and 4 participants had more than 10 years of experience. Fifteen participants reported driving more than 8 h per day, and 1 participant reported driving 6–8 h per day.

Given the modest sample size, the present study should be regarded as an exploratory pilot investigation rather than a confirmatory validation study. The aim was to examine whether a multi-domain human-performance framework could reveal potentially meaningful age- and gender-related differences in safety-relevant driver functional domains.

### 2.2. Human-Centered Multimodal Evaluation Framework

This study adopted a human-centered multimodal framework for characterizing safety-relevant driver functional domains through multiple domains of human performance. Rather than treating individual tests as isolated measurements, the present study organized them into a structured framework comprising self-perception, psychomotor performance, and cognitive–perceptual performance. The framework consisted of three primary domains:

1. Self-perception domain: capturing subjective awareness, behavioral tendencies, and self-reported driving practices.
2. Psychomotor domain: measuring neuromuscular coordination, motor control, and biomechanical performance.
3. Cognitive–perceptual domain: evaluating attention, visual tracking, information processing, and hazard perception.

**Figure 1:**
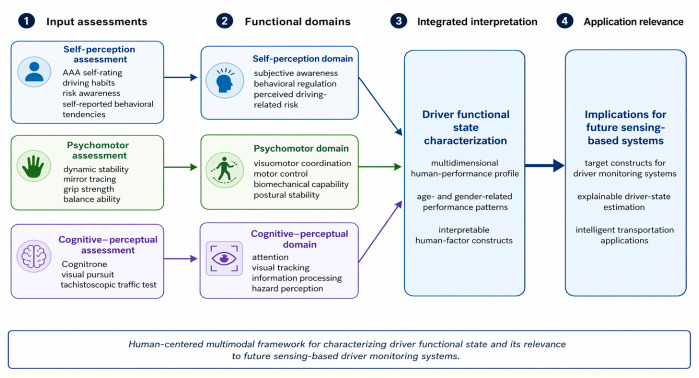
Human-centered multimodal framework proposed in this study. Self-perception, psychomotor, and cognitive–perceptual assessments are organized into three corresponding functional domains, which together provide a structured interpretation of safety-relevant driver functional domains. The framework also illustrates how experimentally measured human-performance variables may serve as interpretable candidate constructs for future sensing-based driver monitoring systems and intelligent transportation applications.

Figure 1 illustrates the proposed human-centered multimodal framework used in the present study. As shown in the figure, the evaluation approach integrates three categories of assessment—self-perception, psychomotor, and cognitive–perceptual performance—and organizes them into corresponding functional domains. These domains jointly support a structured characterization of safety-relevant driver functional domains and provide an interpretable basis for discussing how experimentally measured human-performance variables may inform future sensing-based driver monitoring systems and intelligent transportation applications.

### 2.3. Self-Perception Assessment

Driver self-perception was assessed using a 15-item self-rating questionnaire adapted from the American Automobile Association (AAA) Foundation for Traffic Safety. This instrument was used to capture drivers’ subjective awareness of their own driving habits, rule compliance, perceived limitations, and incident-related behavioral tendencies. In the context of the present study, the questionnaire was treated as a self-perception measure reflecting behavioral awareness and self-regulation, rather than as a stand-alone predictor of actual driving safety.

For items 1–13, responses were scored using a three-level format reflecting the frequency or consistency of the reported behavior (e.g., always/almost always, sometimes, never/almost never). Items 14 and 15 were scored using frequency-based response categories (e.g., none, one or two, three or more). Following the original scoring procedure, weighted scores were calculated by multiplying square-marked items by five and triangle-marked items by three, and then summing the weighted item scores to obtain the total AAA self-rating score.

The total score was interpreted using three profile categories: 0–15 indicating a relatively safe profile, 16–34 indicating a cautionary profile, and ≥35 indicating an unsafe profile. In the present framework, these categories were used only as descriptive self-perception profiles and were not interpreted as validated real-world crash-risk classifications. Instead, the resulting AAA scores were treated as subjective indicators of driving-related self-awareness that could be compared with objective psychomotor and cognitive–perceptual measures across domains.

### 2.4. Psychomotor Assessment

Psychomotor assessment was conducted to characterize visuomotor coordination, motor control, postural stability, and biomechanical capability through standardized task-based measurements. Within the proposed framework, these measures were not interpreted as direct predictors of real-world crash risk or real-time driver state. Rather, they were treated as exploratory performance-based indicators of safety-relevant human functional domains that may help characterize differences in driver-related motor and coordination capacity.

#### 2.4.1. Hand–Eye Coordination Tests

Hand–eye coordination was assessed using two complementary psychomotor tasks: a dynamic stability test and a mirror tracing test. Both tasks required continuous visuomotor integration under constrained or altered visual feedback conditions and were included to capture different aspects of visuomotor performance relevant to coordinated driver responses.

**Figure 2:**
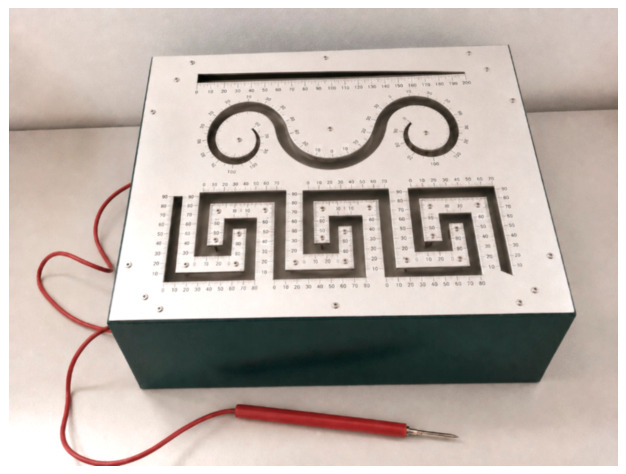
Arm stabilimeter used in the dynamic stability test, in which participants guided a stylus along a narrow track while minimizing contact with the boundaries.

Dynamic Stability Test:

An arm stabilimeter (Model T.K.K. 1211, Takei Scientific Instruments Co., Ltd., Niigata, Japan) [26] was used to evaluate fine motor control and movement stability (Figure 2). Participants were instructed to guide a stylus along a narrow track while minimizing contact with the boundaries. Completion time and the number of boundary-contact errors were recorded as indicators of visuomotor control and hand stability. In the present framework, this task was interpreted as an exploratory indicator of fine motor coordination under visually guided movement constraints.

**Figure 3:**
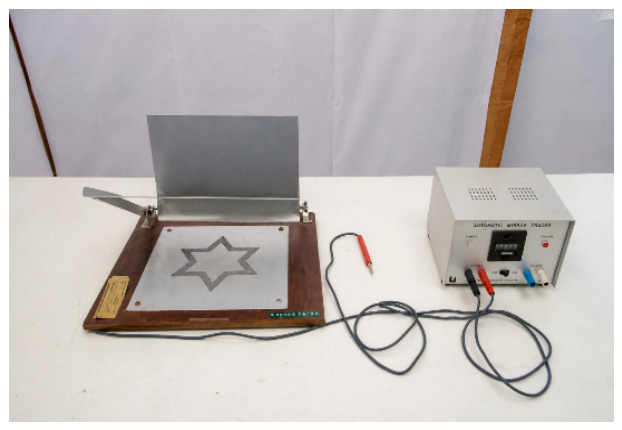
Auto-scoring mirror tracing apparatus used in the mirror tracing test, in which participants traced a figure while viewing only its mirror image.

Mirror Tracing Test:

A mirror tracing apparatus (Model 58024A, Lafayette Instrument Co., Lafayette, IN, USA) [27] was used to assess visuomotor adaptation under reversed visual feedback conditions (Figure 3). Participants were required to trace a geometric figure while viewing only its mirror image. Performance was quantified using completion time and number of errors. This task was included to reflect visuomotor transformation and sensorimotor adaptation processes under altered feedback conditions. Within the proposed framework, it was interpreted as an exploratory indicator of coordination efficiency when visual input and motor output must be continuously recalibrated.

#### 2.4.2. Grip Strength and Balance

Grip strength was measured using a digital hand dynamometer to quantify biomechanical capability. Participants performed two trials for each hand, and the maximum value was recorded. Although grip strength is not a direct measure of driving performance, it was included as a supplementary indicator of upper-limb physical capability within the broader human-factor framework.

Balance ability was evaluated using a single-leg stance test with eyes closed. Participants were instructed to maintain balance for as long as possible, with time recorded in seconds. This task was used to reflect postural control and sensorimotor integration. Similar to grip strength, balance performance was not interpreted as a direct indicator of crash involvement or online driver monitoring status, but rather as a supplementary physical-function measure that may contribute to a broader characterization of driver-related functional stability.

### 2.5. Cognitive–Perceptual Assessment

Cognitive–perceptual performance was evaluated using the Vienna Test System (Schuhfried GmbH, Mödling, Austria), a computerized platform widely used in traffic psychology and human-performance assessment [28]. In the present study, selected Vienna Test System subtests were used to characterize attention-related, visual-tracking, and hazard-perception-related aspects of cognitive–perceptual functioning. These tasks were not treated as direct real-world driving validation measures, but rather as structured laboratory-based indicators of safety-relevant cognitive–perceptual domains.

The Cognitrone test was used to assess sustained attention and concentration under time-constrained conditions. Participants were required to compare abstract figures and make rapid correct-rejection or matching judgments. Performance was quantified primarily through response time and working time measures, which were interpreted as indicators of attention-related processing efficiency.

The visual pursuit test was used to evaluate selective attention and visual tracking efficiency. In this task, participants followed complex visual paths and identified the correct endpoints. Performance measures included number of completed responses and working time. Within the proposed framework, this task was interpreted as an indicator of visual search organization and tracking-related attentional efficiency.

The tachistoscopic traffic test was used to assess rapid traffic-scene perception under brief visual presentation. Participants were required to identify traffic-related elements, such as vehicles, pedestrians, and traffic signals, after short exposure to visual scenes. Accuracy and processing time were recorded. In the present framework, this task was interpreted as an exploratory indicator of time-constrained hazard-perception-related processing.

Taken together, these Vienna Test System subtests served as comparative cognitive–perceptual benchmarks within the broader multimodal framework. Their role in the present study was not to validate a monitoring system or to establish direct driving-performance equivalence, but to provide structured cognitive–perceptual reference measures that could be compared with self-perception and psychomotor performance across domains.

### 2.6. Experimental Procedure

All experiments were conducted in a controlled laboratory environment to ensure consistency of testing conditions. Participants were seated at a standardized viewing distance with a fixed visual angle to the display. Ambient lighting and noise levels were controlled to minimize external interference. The order of tasks was counterbalanced to reduce sequence effects. Rest intervals of at least 10 min were provided between test components to prevent fatigue-related bias. All instruments were calibrated according to the manufacturers’ specifications prior to data collection to ensure measurement reliability.

### 2.7. Data Analysis

Descriptive statistics were first calculated to summarize participant characteristics and performance indicators across the self-perception, psychomotor, and cognitive–perceptual domains. Group differences were then examined using analysis of variance (ANOVA), with age group and gender treated as grouping factors. Separate ANOVA models were conducted for each dependent variable because the outcome measures represented different functional domains and test-specific performance indicators. Statistical significance was set at *p* < 0.05. Effect sizes were reported where appropriate to support the interpretation of group differences beyond statistical significance. Given the small sample size, ANOVA was used as an exploratory screening approach to examine preliminary group-related patterns across predefined age and gender categories, rather than as a confirmatory hypothesis-testing procedure. The use of ANOVA was considered appropriate for the present exploratory purpose because the analyses focused on comparing group means for predefined dependent variables, while the resulting statistical findings were interpreted cautiously in combination with effect sizes, assumption checks, and Holm-adjusted post hoc comparisons.

Prior to conducting ANOVA, the assumptions of approximate normality and homogeneity of variance were examined. The normality of model residuals was assessed using the Shapiro–Wilk test, and homogeneity of variance across groups was evaluated using Levene’s test. Because several outcome variables were bounded or count-like indicators and the subgroup sizes were modest, assumption-testing results were interpreted cautiously rather than used as strict confirmatory criteria. Observations were considered independent because each participant contributed one set of measurements to each analysis. Given the modest sample size and uneven subgroup distribution, the results of assumption testing were interpreted cautiously.

When a significant or potentially meaningful group effect was identified, post hoc pairwise comparisons were conducted where applicable. Holm-adjusted *p*-values were used for post hoc comparisons to reduce the risk of Type I error inflation associated with multiple comparisons. The Holm correction was selected because it controls the family-wise error rate while being less conservative than the Bonferroni correction, which is appropriate for the exploratory nature and modest sample size of the present study. Accordingly, the statistical analyses were intended to identify preliminary group-related patterns rather than provide confirmatory evidence.

## 3. Results

### 3.1. Self-Perception Results

Descriptive statistics and profile interpretations of the American Automobile Association (AAA) self-rating scores, based on 18 participants, are summarized in Table 1. As shown in the table, male drivers exhibited a higher mean AAA self-rating score (27.9) than female drivers (12.9), indicating a greater tendency toward cautionary or unsafe self-perception profiles. In contrast, female drivers showed lower mean scores overall, suggesting relatively safer self-perception profiles.

Age-related differences were also observed in the AAA self-rating results. Drivers aged 30–39 years showed the highest mean score (32.5), followed by those aged 40–49 years (24.0), whereas drivers aged 50–59 years showed the lowest mean score (11.0). Based on the profile interpretation of the AAA scale, the younger group tended to fall within the cautionary or unsafe range, the middle-aged group was predominantly characterized by cautionary profiles, and the older group was more frequently associated with safe or cautionary profiles.

An exploratory ANOVA indicated that the gender-related difference in AAA self-rating scores did not reach conventional statistical significance (*F*(1, 16) = 4.16, *p* = 0.058, $\eta_{p}^{2}$ = 0.21). No statistically significant age-group effect was observed. Given the modest sample size, these findings should be interpreted cautiously and viewed as preliminary group differences in self-perception rather than definitive behavioral-risk classifications.

Within the proposed framework, the AAA self-rating results provide information on subjective awareness of driving behavior, self-regulation, and perceived driving-related risk. Accordingly, the self-perception domain complements the objective psychomotor and cognitive–perceptual measures reported in the following sections and may help identify discrepancies between self-evaluated and performance-based driver-related characteristics.

### 3.2. Psychomotor Performance Results

The psychomotor performance results are summarized in Table 2. Overall, several indicators showed observable group-related patterns across age and gender groups, particularly in visuomotor control and biomechanical capability. However, after considering the exploratory nature of the study, the modest subgroup sizes, and the use of Holm-adjusted post hoc comparisons, these findings should be interpreted with caution.

In the visuomotor control tasks, female drivers required longer completion time than male drivers in the straight path task with the right hand. In the circular path task, male drivers showed more left-hand tracing errors than female drivers. A similar pattern was observed in the square path task, where male drivers and younger drivers showed higher mean numbers of left-hand errors. Older drivers also required longer completion time in the square path task. These findings suggest possible group-related differences in fine motor control and visuomotor coordination; however, because these differences did not remain sufficiently robust after correction for multiple comparisons, they should be interpreted as descriptive patterns rather than definitive demographic effects.

Mirror tracing performance also showed observable gender- and age-related patterns. Female drivers showed higher mean numbers of left-hand mirror-tracing errors and longer right-hand completion time than male drivers. Older drivers also tended to require longer completion time in the right-hand mirror tracing task. These results may suggest that mirror-reversed visuomotor coordination is sensitive to individual differences in psychomotor performance. Nevertheless, these findings should be regarded as exploratory tendencies rather than statistically robust group differences.

In the postural stability category, no prominent demographic differences were observed in balance ability for either the right or left foot. The mean values were generally comparable across gender and age groups, suggesting that balance performance showed limited group-related variation in the present sample.

The most robust psychomotor difference was observed in grip strength. Male drivers demonstrated significantly greater grip strength than female drivers for both the right and left hands, and these differences remained statistically meaningful after Holm-adjusted comparison. This finding indicates that biomechanical capability, particularly hand grip strength, showed the clearest gender-related difference among the psychomotor indicators examined in this study.

Taken together, the psychomotor results indicate that selected visuomotor indicators showed descriptive group-related patterns, whereas grip strength provided the most statistically robust psychomotor finding. Therefore, the psychomotor domain may contribute to the characterization of driver functional profiles, but the observed differences should be interpreted as preliminary evidence requiring validation in larger and more balanced samples.

### 3.3. Cognitive–Perceptual Performance Results

The cognitive–perceptual performance indicators, based on 16 valid participants, are summarized in Table 3. Overall, the results revealed age- and gender-related patterns across measures of sustained attention, visual tracking, and rapid traffic-scene processing. Given the modest sample size, uneven subgroup distribution, and the use of multiple outcome measures, these findings should be interpreted cautiously as exploratory rather than confirmatory group-level effects.

In the Cognitrone test, which reflects sustained attention and response efficiency, older drivers showed slower response performance and longer total working times than younger drivers. Exploratory ANOVA indicated age-group effects for mean time to correct rejection (*F*(2, 13) = 3.92, *p* = 0.047, $\eta_{p}^{2}$ = 0.38) and working time (*F*(2, 13) = 5.00, *p* = 0.025, $\eta_{p}^{2}$ = 0.43), suggesting reduced efficiency in attention-related processing among older participants. However, the corresponding post hoc comparisons did not remain sufficiently robust after Holm correction. Therefore, these findings should be interpreted as descriptive age-related patterns rather than definitive group differences.

In the visual pursuit test, which reflects selective attention and visual tracking efficiency, exploratory ANOVA indicated a gender-related difference in the number of viewed items (*F*(1, 14) = 4.78, *p* = 0.046, $\eta_{p}^{2}$ = 0.25), with female drivers showing a slightly higher number of viewed items. An age-related difference was also observed in total working time (*F*(2, 13) = 3.73, *p* = 0.053, $\eta_{p}^{2}$ = 0.36), suggesting possible group-dependent variation in visual tracking performance. However, the age-related effect was marginal, and the observed gender-related pattern did not remain sufficiently robust after correction for multiple comparisons. Therefore, the visual pursuit results should be interpreted as descriptive patterns rather than statistically robust group differences.

In the tachistoscopic traffic test, which reflects rapid traffic-scene processing under time-constrained conditions, younger drivers achieved more correct responses, whereas older drivers showed a higher number of errors and reduced overall efficiency. Exploratory ANOVA indicated an age-group effect for the number of correct responses (*F*(2, 13) = 4.06, *p* = 0.043, $\eta_{p}^{2}$ = 0.38). A stronger age-group effect was observed for the number of wrong answers (*F*(2, 13) = 8.14, *p* = 0.005, $\eta_{p}^{2}$ = 0.56), indicating that older participants were more likely to make incorrect responses under brief visual presentation conditions. Holm-adjusted post hoc comparisons indicated that the youngest group showed more correct responses than the oldest group, and that the oldest group showed more wrong answers than the middle-aged group. These findings suggest that rapid traffic-scene processing may be more sensitive to age-related functional differences than the other cognitive–perceptual indicators examined in this exploratory sample.

Taken together, the cognitive–perceptual results indicate that sustained attention and visual tracking indicators showed descriptive age- or gender-related patterns, whereas rapid traffic-scene processing provided the most robust age-related finding. These results support the inclusion of cognitive–perceptual measures in the proposed multi-domain driver functional-state assessment framework, while also highlighting the need for cautious interpretation due to the exploratory nature of the study.

**Figure 4:**
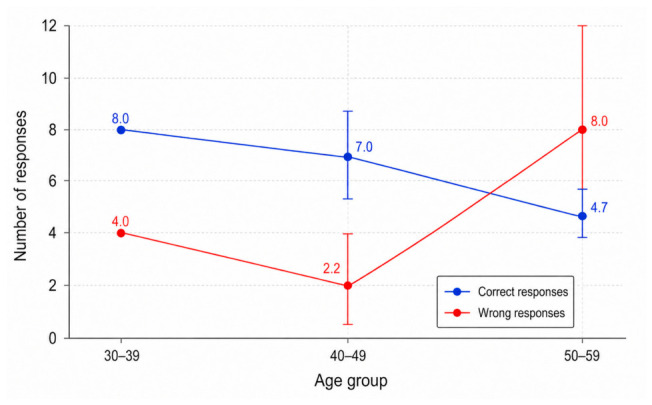
Age-group patterns in selected human-factor performance indicators relevant to driver functional-state assessment. Data points represent group means, and error bars indicate ±1 SD. The figure illustrates descriptive age-related trends and should be interpreted cautiously due to the modest and uneven subgroup sizes.

A cross-domain interpretation of the assessed variables is provided in Table 4. As shown there, the measured human-performance indicators can be interpreted not only as separate task outcomes, but also as functionally meaningful components of safety-relevant driver functional domains that may inform future driver monitoring research. Within the cognitive–perceptual domain, the tachistoscopic traffic test showed the most robust age-related finding after Holm-adjusted post hoc comparisons, particularly for the number of wrong answers under time-constrained traffic-scene presentation. To visualize this result, Figure 4 presents the age-group means and ±1 SD error bars for the number of correct and wrong responses on this task. Given the modest and uneven subgroup sizes, Figure 4 should be interpreted as a descriptive visualization of age-related performance patterns rather than definitive group differences.

### 3.4. Summary of Results

Overall, the results indicate that performance measures derived from self-perception, psychomotor assessment, and cognitive–perceptual assessment were sensitive to selected age- and gender-related differences. Older drivers generally showed slower response performance and lower efficiency in several cognitive–perceptual measures, whereas gender-related differences were mainly reflected in task-dependent performance tendencies across selected psychomotor and visual-tracking assessments.

These findings support the value of multidimensional human-performance measures for exploratory characterization of safety-relevant driver functional domains.

## 4. Discussion

This study proposed a human-centered multimodal framework for characterizing safety-relevant driver functional domains in professional bus drivers by integrating self-perception, psychomotor performance, and cognitive–perceptual assessment. Overall, the findings showed that multidimensional human-performance measures were able to reveal selected age- and gender-related differences and descriptive tendencies, supporting the value of a structured cross-domain approach for examining driver-related functional variability in an exploratory manner. After applying Holm-adjusted post hoc comparisons, the most robust findings were observed in grip strength and tachistoscopic traffic-scene processing, whereas several other indicators should be interpreted as exploratory tendencies rather than definitive demographic effects.

A central contribution of the present study is that it helps clarify what future driver-monitoring research should attempt to interpret beyond surface-level symptom detection alone. In many intelligent transportation applications, driver-monitoring systems are primarily designed to detect relatively direct and observable signs such as reduced vigilance, gaze deviation, or fatigue-related behavior. However, from a human-factors perspective, the more fundamental question is whether such observable signals reflect meaningful differences in underlying driver-related functional domains. The present framework addresses this issue by organizing experimentally measured variables into interpretable domains relevant to driving safety, including self-regulation, visuomotor coordination, attention, visual tracking, and hazard-perception-related processing.

The self-perception results descriptively suggested that male drivers reported higher AAA self-rating scores than female drivers, indicating possible group differences in self-reported driving-related awareness or behavioral tendencies. However, this gender-related difference did not reach conventional statistical significance and should therefore be interpreted cautiously. Rather than supporting definitive behavioral-risk classifications, the AAA findings provide a subjective profile of self-regulation and perceived driving-related risk that may offer complementary information when considered alongside objective performance-based measures.

The psychomotor findings suggested that different driver groups may exhibit different visuomotor and physical performance tendencies across task conditions. Female drivers generally required longer completion times in selected tracing tasks, whereas male drivers showed more errors in some dynamic stability conditions. At the same time, grip strength showed the clearest and most stable gender-related difference among the psychomotor indicators, with male drivers demonstrating significantly greater grip strength in both hands after Holm-adjusted comparison. These findings should not be interpreted as fixed demographic performance profiles. Instead, they suggest that task-based psychomotor measures may capture different aspects of movement control, coordination efficiency, and physical functional capacity, which may vary across individuals and groups in different ways.

Mirror tracing results should be interpreted particularly cautiously. Although some metrics suggested group-dependent differences in completion time or error tendency, the observed effects were not equally stable across all mirror-tracing indicators and did not remain sufficiently robust after considering multiple comparisons. Accordingly, the mirror-tracing findings are better understood as exploratory evidence of possible differences in visuomotor adaptation under altered visual feedback rather than as a basis for strong demographic conclusions.

Age-related patterns were observed in the cognitive–perceptual domain, although their robustness differed across tasks. In the Cognitrone test, older drivers generally showed slower response performance and longer working times, suggesting reduced efficiency in sustained attention and response-related processing. These patterns are broadly consistent with prior human-factors literature indicating that aging may be associated with slower information processing and reduced attentional efficiency. However, after Holm-adjusted post hoc comparisons, these findings should be interpreted as descriptive age-related patterns rather than definitive group differences. The contribution of the present study is therefore not simply to reconfirm that age-related differences exist, but to show how such differences may emerge across multiple assessed domains within a common interpretive framework.

The visual pursuit results suggested more limited and less stable group differences than the other cognitive–perceptual measures. Female drivers showed a slightly higher number of viewed items, whereas younger drivers tended to complete the task more quickly. However, these patterns were relatively modest and were not robust after correction for multiple comparisons. In the present framework, the visual pursuit task remains useful because it contributes a structured reference measure of visual tracking and search efficiency, even if the demographic effects observed here were not uniformly strong.

Among the cognitive–perceptual measures, the tachistoscopic traffic test provided the clearest age-related finding after Holm-adjusted post hoc comparisons. Younger drivers achieved more correct responses, whereas older drivers showed a higher number of wrong answers, indicating reduced efficiency under time-constrained visual presentation conditions. This pattern is important because it suggests that age-related differences in this domain were not limited to slower responding but also involved a higher error tendency during rapid traffic-scene recognition. In the present framework, this task therefore serves as a meaningful candidate indicator of hazard-perception-related processing under constrained perceptual conditions.

The Vienna Test System results also served an important comparative role in the present study. The selected subtests related to sustained attention, visual pursuit, and tachistoscopic traffic-scene processing provided structured laboratory-based benchmarks within the broader multimodal framework. Their role here was not to validate a monitoring system or to demonstrate direct equivalence with actual on-road performance. Instead, they offered comparative cognitive–perceptual reference measures that helped contextualize how performance may vary across driver groups and how such variation may be interpreted alongside self-perception and psychomotor performance.

One of the more meaningful observations in the present study was the possible mismatch between subjective self-perception and objective performance. Some drivers with relatively safer self-perception profiles nonetheless showed weaker psychomotor or cognitive–perceptual performance patterns. Although these observations remain exploratory, they suggest that subjective confidence or self-evaluated safety may not always align with performance-based indicators. This kind of discrepancy may be important in future driver evaluation research because it highlights the possibility that some safety-relevant limitations are not fully captured by self-report alone.

Taken together, the findings support the use of a broader human-factor perspective when discussing future driver monitoring. The present study did not attempt to develop or validate a real-time sensing system, nor did it establish direct predictive rules for intelligent transportation applications. Instead, its contribution lies in clarifying which experimentally measured human-performance variables may represent candidate safety-relevant functional domains and why those domains may matter. In this sense, the value of the proposed framework is not limited to detecting visible symptoms such as drowsiness alone, but extends to the interpretation of monitored driver-related signals or behaviors in terms of underlying human functional meaning.

Building on the cross-domain interpretation summarized in Table 4, the measured indicators should be understood not only as isolated task outcomes, but also as candidate components of safety-relevant driver functional domains. This interpretation provides the basis for considering how laboratory-based human-performance variables may be conceptually linked to future driver-monitoring observables.

To further clarify the relevance of the present framework to future driver-monitoring research, Table 5 provides an illustrative mapping between the assessed human-performance domains and possible observable driver-related behaviors, signals, or sensing modalities. Specifically, the table links self-perception profiles to long-term behavioral tendencies or self-regulation-related constructs; psychomotor indicators to possible vehicle-control behaviors such as steering smoothness, correction delay, manual control stability, or adaptation to indirect visual feedback; and cognitive–perceptual indicators to gaze behavior, visual search efficiency, response delay, hazard-detection-related performance, and traffic-response readiness. This mapping is consistent with recent driver-monitoring studies that have used physiological signals, eye-tracking metrics, multimodal physiological datasets, and camera-based facial or gaze-related features to infer driver attention, fatigue, cognitive load, or behavioral state in simulated or driving-related contexts [1,2,3,4]. In this way, Table 5 extends the cross-domain interpretation in Table 4 by showing how laboratory-based human-performance variables may be translated into candidate constructs for future sensing-based driver-monitoring research.

However, the mapping in Table 5 should be interpreted as conceptual and exploratory rather than as a validated sensing model. The present study did not collect real-time in-vehicle signals, validate sensor-based monitoring outputs, or establish predictive links between the assessed laboratory variables and on-road driving behavior. Therefore, Table 5 is intended to clarify the possible relevance of the proposed functional domains to future driver-monitoring observables, not to define operational ADAS intervention rules, real-time monitoring thresholds, or finalized sensing outputs.

Compared with conventional symptom-based or single-indicator driver-monitoring approaches, the proposed framework does not aim to directly classify driver state or predict on-road driving outcomes. Instead, it provides a conceptual benchmark for organizing laboratory-based human-performance measures into interpretable functional domains. Conventional driver-monitoring approaches often focus on observable indicators such as fatigue-related behaviors, gaze deviation, response delay, or other directly measurable behavioral signals. In contrast, the present framework emphasizes how such observable behaviors may be conceptually linked to underlying self-perceptual, psychomotor, and cognitive–perceptual domains. This comparison highlights the potential value of the proposed framework as an interpretive structure for future sensing-based driver-monitoring research, while also acknowledging that quantitative validation and predictive evaluation require larger samples and simulator-based or on-road driving data.

Rather than relying only on observable or data-driven features, the present study emphasizes the importance of linking monitored indicators to interpretable functional domains so that driver-related assessment can be discussed in clearer safety-related terms. The proposed framework may also have practical implications for intelligent transportation and occupational driver evaluation. It may support more structured driver assessment, offer interpretable reference dimensions for targeted retraining, and help frame more individualized approaches to professional-driver management. In particular, a function-oriented perspective may help future intelligent assistance systems evaluate not only whether a driver shows an obvious symptom, but also whether the driver’s responses remain sufficiently timely, coordinated, and hazard-sensitive for safe operation.

Several limitations should be acknowledged. First, the sample size was modest, and the subgroup comparisons were based on relatively small and uneven numbers of participants, particularly across age groups. This may have limited the statistical power to detect small-to-moderate group differences. Therefore, non-significant findings should not be interpreted as evidence of no effect, and statistically significant findings should be regarded as preliminary evidence requiring confirmation in larger and more balanced samples. Second, although Holm correction was applied to reduce the risk of Type I error inflation in post hoc comparisons, the number of outcome measures and the exploratory nature of the analyses still require cautious interpretation. Third, although ANOVA assumptions were examined prior to analysis, the modest sample size and uneven subgroup distribution may limit the reliability of formal assumption tests. Therefore, both the assumption testing results and the statistical findings should be interpreted cautiously.

Additional limitations are related to the study design and measurement scope. The study was conducted in a controlled laboratory setting using structured assessment tasks. As a result, the measured variables are more appropriately interpreted as candidate indicators of safety-relevant human functional domains than as direct measures of actual on-road driver state or validated real-time monitoring outputs. Although the proposed framework is discussed in relation to future driver-monitoring and intelligent transportation applications, the present study did not implement or validate a sensing system, did not include simulator-based or on-road driving tasks, and did not establish predictive relationships between the measured variables and real-world driving outcomes. In addition, some psychomotor and physical measures, such as grip strength, balance, dynamic stability, and mirror tracing, should be interpreted as supplementary and indirect indicators of driver-related functional capacity rather than direct predictors of crash involvement or operational driving safety.

Finally, although professional driving experience and daily driving hours were summarized as occupational background variables, several occupational and contextual factors were not systematically incorporated in the present design. Detailed variables such as annual mileage, work schedules, shift patterns, sleep condition, recent fatigue status, and recent driving load may influence psychomotor and cognitive–perceptual performance, but they were not fully controlled in this exploratory study. The use of module-specific valid samples, with 18 participants for the self-perception questionnaire and 16 participants for the performance-based modules, should also be taken into account when interpreting cross-domain comparisons. Future studies should include larger and more diverse driver samples, conduct a priori power analysis, incorporate simulator-based or real-world driving validation, and examine how these candidate human-performance domains relate to observable in-vehicle signals such as gaze behavior, steering behavior, pedal responses, fatigue-related indicators, heart-rate-related measures, electrodermal activity, and other physiological or behavioral sensing outputs.

Overall, the present findings support the potential usefulness of a multi-domain human-factor assessment framework for characterizing professional bus drivers’ functional state. The strongest empirical support was observed for grip strength and tachistoscopic traffic-scene processing, while other measures provided exploratory information about possible group-related tendencies. These findings suggest that a multi-domain approach may offer a more comprehensive understanding of driver functional profiles than single-indicator assessments. With further validation, such a framework may contribute to future driver-monitoring and adaptive ADAS research by providing preliminary human-factor evidence for individualized driver-state interpretation and intervention design.

## 5. Conclusions

This study proposed a human-centered multimodal framework for characterizing safety-relevant driver functional domains in professional bus drivers. By integrating self-perception, psychomotor performance, and cognitive–perceptual assessment, the proposed framework provides a structured approach for interpreting laboratory-based human-performance indicators as candidate functional domains relevant to future driver-monitoring research.

The empirical findings showed that selected age- and gender-related differences and descriptive tendencies were observable across the assessed domains. After applying Holm-adjusted post hoc comparisons, the most robust findings were observed in grip strength and tachistoscopic traffic-scene processing. Specifically, grip strength showed the clearest gender-related difference within the psychomotor domain, whereas the tachistoscopic traffic test showed the clearest age-related pattern within the cognitive–perceptual domain, particularly in terms of increased error tendency under time-constrained traffic-scene presentation. Other indicators, including dynamic stability, mirror tracing, Cognitrone measures, and visual pursuit measures, provided exploratory information about possible group-related tendencies but should not be interpreted as definitive demographic effects.

The main contribution of this study lies in its cross-domain interpretive structure rather than in demonstrating that every individual indicator produced statistically robust group differences. By organizing self-perceptual, psychomotor, and cognitive–perceptual measures into functionally meaningful driver-related domains, the framework may help clarify how experimentally measured human-performance variables can be interpreted as candidate constructs for future sensing-based driver monitoring. This perspective supports a more explainable and human-centered approach to driver-state interpretation beyond surface-level symptom detection alone.

The present study should be interpreted as an exploratory pilot investigation. The modest sample size, uneven subgroup distribution, and laboratory-based assessment design limit the generalizability of the findings. In addition, this study did not implement a real-time sensing system, validate sensor-based monitoring outputs, or establish direct predictive relationships between the assessed variables and on-road driving performance. Therefore, the proposed framework should not be interpreted as a finalized driver-monitoring model, operational sensing model, or adaptive ADAS control strategy.

Future studies should recruit larger and more balanced samples, conduct a priori power analysis, incorporate occupational and contextual variables such as annual mileage, work schedules, shift patterns, sleep condition, and recent fatigue status, and validate the proposed functional domains using simulator-based or on-road driving data. Further research should also examine how multi-domain human-performance profiles can be integrated with observable in-vehicle signals, such as gaze behavior, steering behavior, pedal responses, fatigue-related indicators, and physiological measures. With further validation, the proposed framework may provide a useful human-factor basis for individualized driver-state interpretation and future adaptive ADAS intervention design.

## Figures and Tables

**Table 1:** Summary of AAA self-rating scores and profile interpretation by gender and age group.

Grouping Factor	Subgroup	Mean AAA Score	SD	Min	Max	Safe (0–15)	Caution (16–34)	Unsafe (≥35)
Gender	Male	27.9	17.8	5	70	2	5	4
	Female	12.9	9.5	0	27	4	3	0
Age group (yr)	30–39	32.5	7.8	27	38	0	1	1
	40–49	24.0	18.1	3	70	4	5	3
	50–59	11.0	9.9	0	22	2	2	0

Notes: AAA self-rating data were based on 18 participants. Higher scores indicate less favorable self-perception profiles. Profile interpretation was based on the following score ranges: 0–15, safe; 16–34, caution; ≥35, unsafe.

**Table 2:** Summary of representative psychomotor performance indicators by functional category.

Functional Category	Representative Indicator	Male	Female	30–39	40–49	50–59	Main Observed Pattern
Visuomotor control	Straight path, right-hand time (s)	4.6	6.6	2.5	5.8	5.5	Female drivers required longer completion time
	Circular path, left-hand mistakes	5.3	1.8	7.0	3.0	5.1	Male drivers showed more tracing errors
	Square path, left-hand mistakes	11.6	7.2	17.0	8.8	9.2	Male and younger drivers showed more left-hand errors
	Square path, left-hand time (s)	19.6	16.4	14.5	16.1	26.2	Older drivers required substantially longer completion time
	Mirror tracing, left-hand mistakes	23.0	77.8	27.8	45.2	47.2	Female drivers showed more mirror-tracing errors
	Mirror tracing, right-hand time (s)	97.0	140.4	71.0	105.7	153.4	Female and older drivers required longer completion time
Postural stability	Balance ability, right foot (s)	15.0	15.0	14.1	14.2	17.6	No prominent demographic differences were observed
	Balance ability, left foot (s)	11.3	14.2	5.2	13.4	13.5	No prominent demographic differences were observed
Biomechanical capability	Grip strength, right hand (kg)	32.5 **	21.2	32.5	26.7	30.0	Male drivers showed significantly greater grip strength after Holm correction
	Grip strength, left hand (kg)	38.0 **	23.0	36.0	31.2	33.5	Male drivers showed significantly greater grip strength after Holm correction

Notes: Values are presented as group means. Significance markers indicate Holm-adjusted results where applicable. Unmarked group differences are presented as descriptive patterns and should not be interpreted as statistically significant. ** *p* < 0.01. Psychomotor performance indicators were based on 16 valid participants.

**Table 3:** Summary of cognitive–perceptual performance indicators and their functional meaning.

Functional Meaning	Indicator	Male	Female	30–39	40–49	50–59	Main Observed Pattern
Cognitrone test (Sustained attention and response efficiency)	Mean time to correct reject (s)	2.6	2.8	1.8	2.5	3.5	Older drivers showed slower correct-rejection responses
	Mean time of hit (s)	2.1	2.0	1.6	1.9	2.6	Response speed decreased with age
	Working time (s)	144.8	145.0	104.5	135.0	189.8	Older drivers required longer total working time
Visual pursuit test (Selective attention and visual tracking efficiency)	Number of answers	18.1	19.0	18.0	18.3	19.0	Female drivers showed a slightly higher response count
	Mean time of correct answer (s)	12.3	10.8	16.5	11.2	10.8	Response-time pattern differed across groups
	Working time (s)	75.6	85.0	58.5	82.2	81.8	Younger drivers completed the task faster, whereas female drivers required longer total time
Tachistoscopic traffic test (Rapid hazard perception and traffic-scene processing)	Number of correct answers	6.8	6.2	8.0 *	7.0	4.8	Younger drivers achieved more correct responses
	Number of wrong answers	4.4	3.2	4.0	2.3	8.0 **	Older drivers showed a higher error tendency
	Working time (s)	156.1	153.8	115.5	145.4	199.8	Older drivers required substantially longer processing time

Notes: Values are presented as group means. Significance markers indicate Holm-adjusted post hoc comparisons where applicable. Unmarked group differences are presented as descriptive patterns and should not be interpreted as statistically significant. * *p* < 0.05, ** *p* < 0.01. Cognitive–perceptual performance indicators were based on 16 valid participants.

**Table 4:** Cross-domain interpretation of assessed variables within the proposed human-centered multimodal framework.

Domain	Assessment/Variable	Functional Interpretation	Main Demographic Pattern Observed in This Study	Potential Relevance to Future Sensing-Based Systems
Self- perception	AAA self-rating	Reflects subjective awareness of driving behavior, self-regulation, and perceived driving-related risk	Male drivers descriptively showed higher self-rating scores, suggesting possible group differences in self-perceived driving-related awareness or behavioral tendencies	May inform behavioral-risk profiling and self-awareness-related driver-state constructs
Psychomotor	Dynamic stability test (mistakes and completion time)	Indicates fine motor control, visuomotor coordination, and movement stability under constrained tracing conditions	Female drivers generally required longer completion times, whereas male drivers showed more errors in selected tracing conditions; older drivers tended to require more time	May provide interpretable proxies for motor coordination and psychomotor control in human-centered driver monitoring
	Mirror tracing test (mistakes and completion time)	Reflects visuomotor adaptation, sensorimotor transformation, and coordination under altered visual feedback	Female drivers showed longer completion times and higher error rates; older drivers also tended to require longer completion times	May serve as a target construct related to visuomotor adaptability and sensorimotor performance
	Balance ability	Represents postural stability and sensorimotor integration	No prominent demographic differences were observed under the present testing condition	May contribute to broader characterization of physical functional stability, although its sensitivity appeared limited in this sample
	Grip strength	Reflects biomechanical capability and upper-limb physical strength	Male drivers demonstrated substantially greater grip strength than female drivers; age-related differences were less pronounced	May support physical-capability profiling as part of multimodal driver-state characterization
Cognitive–perceptual	Cognitrone test	Represents sustained attention, concentration, and response efficiency	Older drivers showed slower response performance and longer working times, indicating reduced efficiency in attention-related processing	May inform interpretable cognitive-state constructs relevant to alertness and attention monitoring
	Visual pursuit test	Reflects selective attention, visual orientation, and tracking efficiency in complex visual environments	Female drivers showed a slightly higher number of viewed items, whereas younger drivers tended to complete the task faster; however, these patterns should be interpreted cautiously	May support sensing-related constructs associated with visual tracking behavior and attentional efficiency
Cognitive–perceptual	Tachistoscopic traffic test	Represents rapid hazard perception, traffic-scene processing, and time-constrained visual recognition	Younger drivers achieved more correct responses, whereas older drivers showed a higher error tendency, indicating reduced efficiency in rapid traffic-scene processing	May serve as a meaningful candidate construct for future sensing-based estimation of hazard-detection capability and traffic-response readiness

Notes: This table provides an interpretive summary of how the assessed self-perception, psychomotor, and cognitive–perceptual variables may be understood as candidate safety-relevant driver functional domains within the proposed framework. The interpretations are intended to be conceptual and exploratory rather than validated real-world monitoring classifications.

**Table 5:** Illustrative mapping between assessed human-performance domains and possible future driver-monitoring observables.

Human-Performance Domain/Assessed Variable	Functional Meaning in the Present Study	Possible Observable Driver-Related Behaviors or Signals in Future Monitoring Contexts	Candidate Sensing Modality or Data Source	Important Limitation of Interpretation
AAA self-rating/self-perception profile	Subjective awareness of driving behavior, self-regulation, and perceived driving-related risk	No direct real-time observable equivalent; may relate indirectly to long-term behavioral tendencies, self-regulation, or mismatch between self-perception and objective behavior	Periodic self-report, driver profiling, longitudinal behavioral records	Not directly measurable as a real-time in-vehicle signal; should not be interpreted as a direct monitoring output
Dynamic stability test	Fine motor control, visuomotor coordination, and movement stability under constrained conditions	Variability in steering smoothness, delayed correction behavior, reduced manual control stability	Steering-wheel input signals, lane-keeping telemetry, vehicle-control traces	Laboratory tracing performance is not equivalent to actual vehicle control and should be interpreted only as an indirect candidate construct
Mirror tracing test	Visuomotor transformation and sensorimotor adaptation under altered visual feedback	Difficulty adapting to indirect visual inputs, delayed mirror-related responses, possible inefficiency in visually guided maneuvering	Eye-tracking, visual-orientation behavior under indirect feedback, camera-based head/gaze monitoring, steering response traces	The task does not replicate real driving maneuvers and should not be treated as a validated driving surrogate
Grip strength	Upper-limb biomechanical capability and physical functional capacity	Reduced physical-response capability or fatigue-related decline in forceful manual operation	Wearable sensing, grip-force devices, steering interaction metrics	Not a direct predictor of driving safety; best interpreted as a supplementary physical-capacity indicator
Balance ability	Postural stability and sensorimotor integration	No direct in-vehicle equivalent under ordinary driving; may relate only indirectly to broader physical functional stability	Wearable inertial sensing, posture-related monitoring in specialized contexts	Limited relevance to standard in-vehicle monitoring; should be treated as a supplementary and indirect measure
Cognitrone test	Sustained attention, concentration, and response efficiency	Slower response initiation, delayed decision-related behavior, reduced attentional efficiency	Eye-tracking, response-latency measures, steering/braking response timing, physiological monitoring	Laboratory cognitive performance does not directly validate real-time attention monitoring in actual driving
Visual pursuit test	Selective attention, visual search organization, and tracking efficiency	Less efficient gaze allocation, fragmented scanning patterns, reduced visual-tracking continuity	Eye-tracking, gaze-path analysis, camera-based visual behavior monitoring	The task provides an indirect reference measure and not a direct representation of on-road gaze behavior
Tachistoscopic traffic test	Time-constrained hazard-perception-related processing and traffic-scene recognition	Delayed hazard detection, slower reaction to traffic-relevant events, reduced scene-processing efficiency	Eye-tracking, pedal response timing, braking reaction measures, event-based vehicle telemetry	The task reflects laboratory-based rapid scene processing and does not directly establish real-world hazard-response validity

## Data Availability

The data presented in this study are not publicly available due to privacy and ethical restrictions involving human participants. The data may be made available from the corresponding author upon reasonable request.
